# Renal epithelial cells retain primary cilia during human acute renal allograft rejection injury

**DOI:** 10.1186/s13104-019-4738-6

**Published:** 2019-11-01

**Authors:** Elizabeth Verghese, Luciano G. Martelotto, Jason E. Cain, Timothy M. Williams, Andrea F. Wise, Prudence A. Hill, Robyn G. Langham, D. Neil Watkins, Sharon D. Ricardo, James A. Deane

**Affiliations:** 1 Biomedical and Health Sciences, Victoria University, St Albans, Australia; 2 Centre for Cancer Research, Hudson Institute of Medical Research, Clayton, Australia; 3 Present Address: Centre for Cancer Research, VCCC, University of Melbourne, Melbourne, Australia; 4 Department of Anatomy and Developmental Biology, Monash University, Clayton, Australia; 5 Department of Anatomical Pathology, St Vincent’s Hospital, Melbourne, Australia; 6 Department of Nephrology, St Vincent’s Hospital, Melbourne, VIC Australia; 7 Present Address: Monash Rural Health, Monash University, Clayton, VIC Australia; 8 The Kinghorn Cancer Centre, Garvan Institute of Medical Research, Darlinghurst, NSW Australia; 9 St. Vincent’s Clinical School, Faculty of Medicine, University of New South Wales Sydney, Darlinghurst, NSW Australia; 10 Department of Obstetrics and Gynaecology, Monash University, Clayton, Australia

**Keywords:** Hedgehog signaling, Primary cilia, Rejection, Renal allograft

## Abstract

**Objectives:**

Primary cilia are sensory organelles which co-ordinate several developmental/repair pathways including hedgehog signalling. Studies of human renal allografts suffering acute tubular necrosis have shown that length of primary cilia borne by epithelial cells doubles throughout the nephron and collecting duct, and then normalises as renal function returns. Conversely the loss of primary cilia has been reported in chronic allograft rejection and linked to defective hedgehog signalling. We investigated the fate of primary cilia in renal allografts suffering acute rejection.

**Results:**

Here we observed that in renal allografts undergoing acute rejection, primary cilia were retained, with their length increasing 1 week after transplantation and remaining elevated. We used a mouse model of acute renal injury to demonstrate that elongated renal primary cilia in the injured renal tubule show evidence of smoothened accumulation, a biomarker for activation of hedgehog signalling. We conclude that primary cilium-mediated activation of hedgehog signalling is still possible during the acute phase of renal allograft rejection.

## Introduction

Renal primary cilia are sensory organelles that co-ordinate signalling pathways involved in proliferation and differentiation, including hedgehog (Hh) and wingless (Wnt) [1]. Canonical Hh activation requires the translocation of the Hh pathway component smoothened (Smo) to the primary cilium [2]. Previous studies have shown that cilium length on renal epithelial cells increases and then normalises with the return of graft function in mouse models of ischemia/reperfusion injury and human renal allografts with acute tubular necrosis [3, 4]. In contrast, studies of a rat model of chronic renal allograft rejection reported the loss of renal primary cilia on epithelial cells and implicated this in the dysregulation of hedgehog signalling contributing to fibrosis [5]. However, little is known about the behaviour of primary cilia and the pathways they regulate in the early acute phase of human renal allograft rejection.

Renal allograft rejection occurs when the recipient’s immune system mounts an immune response against a non-self renal tissue and can destroy a graft. Here we examined renal primary cilia and graft function (assessed by serum creatinine and urine output) in serial biopsies from human renal allografts suffering acute rejection controlled by immunosuppressive drugs. We also explored primary-cilium mediated hedgehog signalling in the context of acute renal injury using a mouse ischemia/reperfusion model.

## Main text

### Methods

#### Measuring primary cilia in renal allograft biopsy samples

Tissue was obtained from needle biopsies taken from human renal allografts suffering acute rejection. The use of biopsy material was approved by the St Vincent hospital Human Ethics committee. Allograft recipients were on standard triple immunosuppression therapy of cyclosporin, mycophenolate mofetil and prednisolone. Paraffin embedded biopsies were obtained between 0 and 40 days post transplantation. Rejection was assessed from two needle biopsy series by an experienced pathologist (PAH) and one was categorized as acute cellular rejection and one as antibody-mediated rejection. Acute rejection was diagnosed by histology and C4d immunostaining. The type and severity of rejection was rated using the Banff scale [refer to Transplantation: November 2018, Volume 102, Issue 11, p 1795–1814]. Graft function data (serum creatinine, urine output (to a maximum of 2 L), and pathology reports) were obtained for each allograft biopsy series.

Primary cilia were visualized and measured as previously described [3]. For each patient biopsy sample multiple sections were examined and 50 proximal tubule and 50 distal tubule/collecting duct cilia were measured.

Cilium length data were analyzed using a one-way ANOVA with an accompanying Tukey’s post hoc test performing intergroup comparisons. Statistically significant differences within segments examined were defined as *p* < 0.05 Values are expressed as mean ± SEM.

#### Smoothened immunolocalization in injured mouse renal tubules

Immunostaining for the Hh signaling pathway component Smo was conducted in mouse kidneys (n = 3 for sham and IR) due to the need to use fixed and frozen material that was not available for human biopsy series.

Mouse studies were approved in advance by a Monash University Animal Ethics Committee and were performed in accordance with the Australian Code of Practice for the Care and Use of Animals for Scientific Purposes. The induction of renal ischemia/reperfusion injury and kidney collection was as described previously [6]. Kidneys from mice that underwent sham surgery were used as controls. Kidneys were perfusion fixed with 4% paraformaldehyde in PBS, cryoprotected in 30% sucrose in PBS and frozen in OCT medium for sectioning.

Localization of Smo to primary cilia in sections was as described previously [7]. Primary cilia were stained using primary antibody to α-acetylated tubulin (1:500) to AlexaFluor-568 (1:1000) and Smo colocalization detected using an antibody to Smo (LifeSpan) (1:100) conjugated to AlexaFluor-488A (1:1000). Antibodies were conjugated using Mix-n-Stain (Biotium).

### Results

**Fig. 1:**
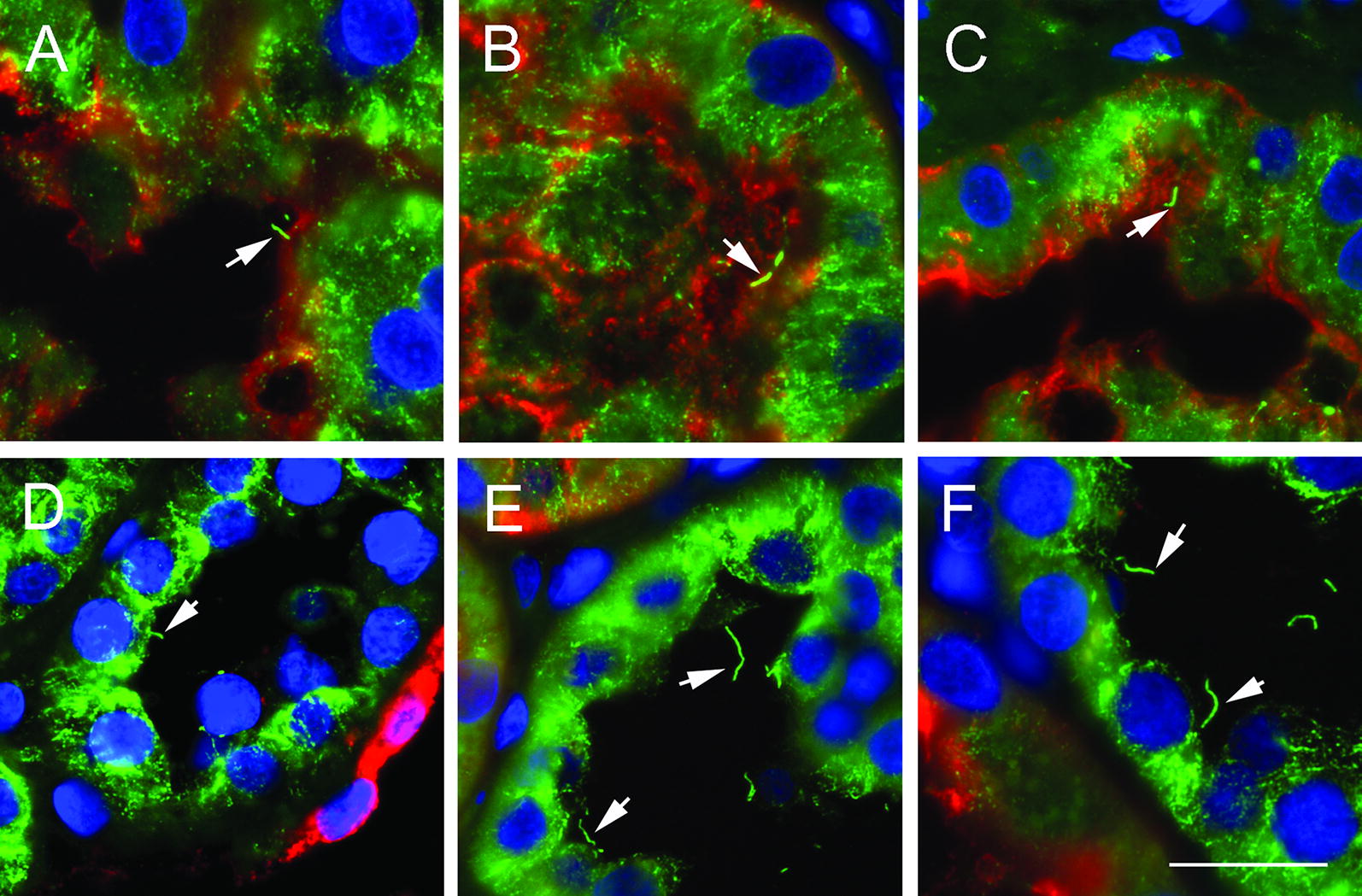
Primary cilia in biopsies from renal allografts suffering acute rejection injury. Representative images from biopsy samples from an allograft suffering antibody-mediated rejection on the day of transplantation (**A**, **D**) and after 9 days (**B**, **E**) and 35 days (**C**, **F**). Renal cilia (arrows) are stained with anti-acetylated α-tubulin (green), the proximal tubule of the brush border with anti-aquaporin-1 (red) and nuclei with DAPI (blue). Examples of proximal tubule are shown in **A**–**C** and the distal tubule/collecting duct cilia in **D**–**F**. Scale bar in F = 20 μm and **A**–**E** are at the same magnification

**Fig. 2:**
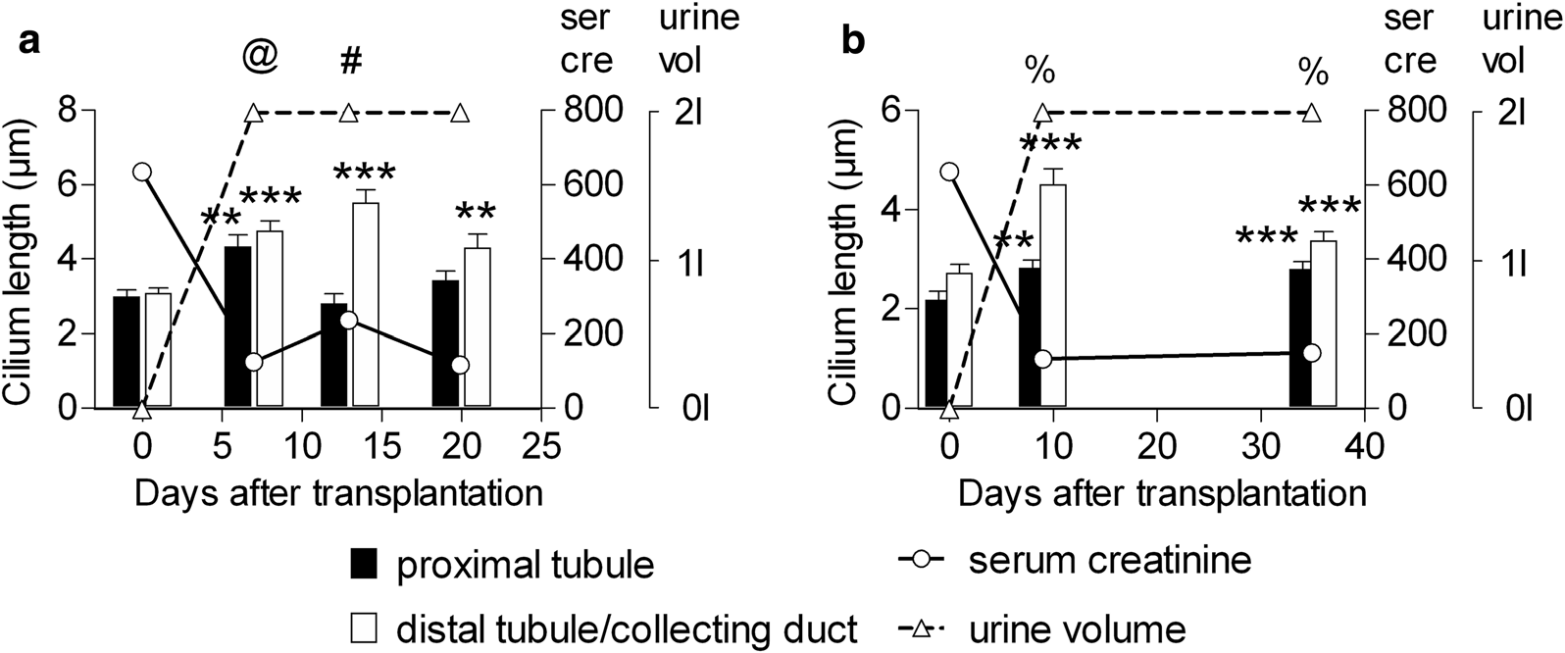
Renal primary cilium elongation during allograft rejection. Quantification of cilium length in an allograft suffering acute cellular rejection (**a**) and an allograft suffering antibody-mediated rejection (**b**). Bars show mean ± SEM for 50 cilia. **p < 0.01 and ***p < 0.001 relative to day zero for this segment as assessed by one way ANOVA with Dunnett’s test. The type and severity of rejection that each allograft underwent is indicated using the Banff Scale. ^@^Banff IA rejection, ^#^Banff IIA rejection, ^%^antibody mediated rejection Grade II. Serum Creatinine (solid line) is shown as µmol/L and urine output (dashed line) to a maximum of 2 L

Primary cilium length was assessed in the aquaporin-1 positive proximal and aquaporin-1 negative remaining segments of the nephron in two biopsy series from human renal allografts undergoing rejection (Figs. 1 and 2). Cilium length on the day of transplantation for both series was 2–3 μm throughout the nephron. Primary cilia were not lost from epithelial cells upon the onset of rejection injury, rather their length increased in both biopsy series examined (Fig. 2). Cilium elongation was most prominent outside the proximal tubule (distal tubule and collecting duct). Renal function, as measured by increasing urine production and falling serum creatinine, recovered and was maintained in the period spanning biopsies (Fig. 2). This suggests a degree of repair/recovery in the allografts.

**Fig. 3:**
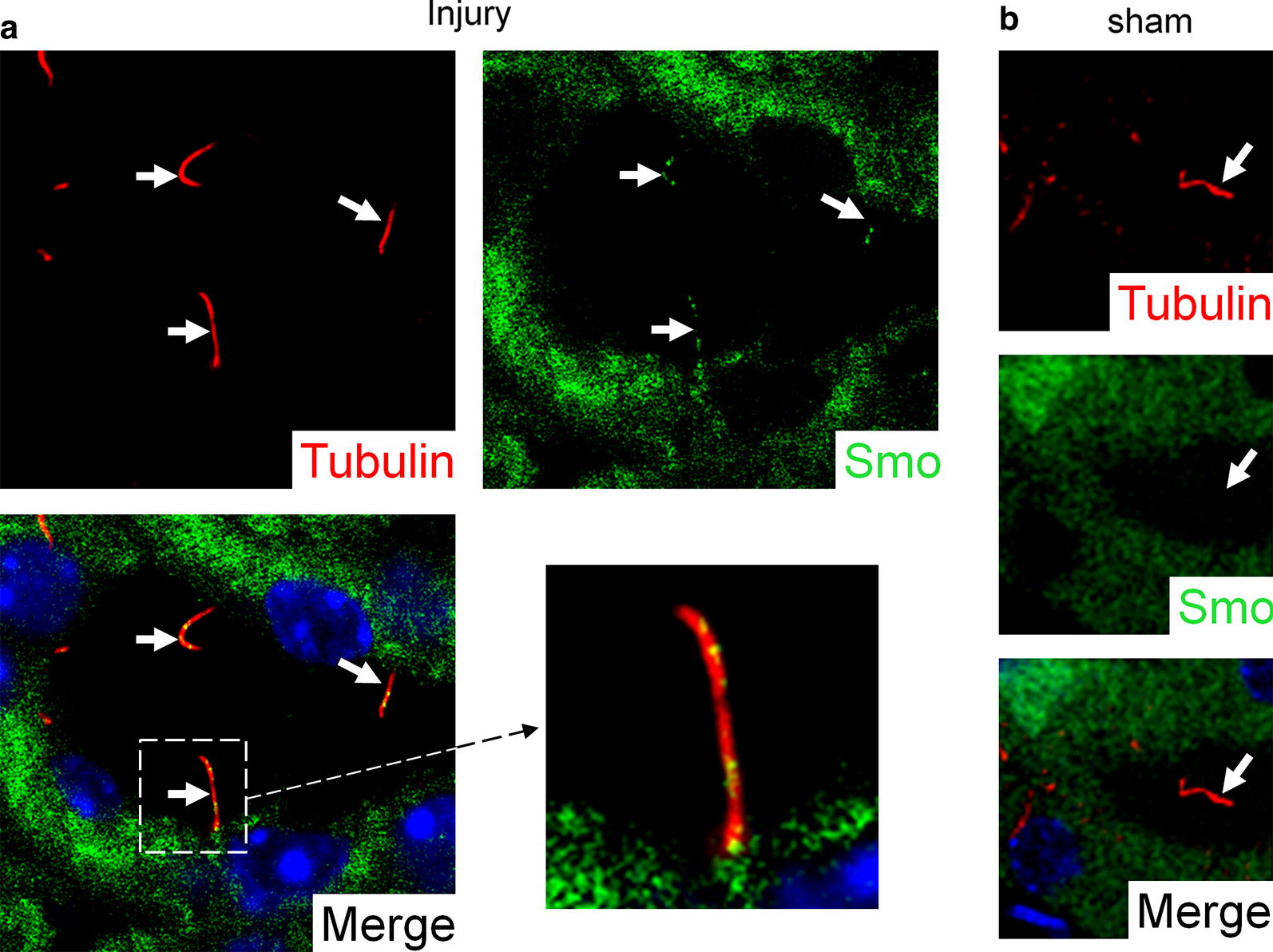
Smoothened accumulation in the primary cilia of mouse kidneys with ischemia/reperfusion injury. Representative images of renal primary cilia (arrows) from mice (n = 3 for sham and IR) are stained with anti-acetylated α-tubulin (red). Smoothened (green) localises to the primary cilia of injured kidney (**a**), but not to the primary cilia of uninjured sham kidney (**b**). Nuclei (blue) are stained with DAPI in merged images

Primary cilia borne by epithelial cells in ischemia/reperfusion injured mouse kidney were elongated and showed punctate accumulations of Smo (Fig. 3a). In contrast, Smo was not localized to primary cilia in control sham surgery kidney (Fig. 3b).

### Discussion

We investigated the fate of primary cilia during rejection in biopsy series from human renal allografts suffering acute rejection. Primary cilia were maintained on epithelial cells of the renal tubule and collecting duct and became longer with the onset of rejection injury.

These findings are in keeping with our previous observations that renal injury causes the elongation of primary cilia on epithelial cells throughout the kidney [4]. We and others have speculated that this is an adaption that increases the sensitivity of primary cilium-mediated signaling in the injured kidney [3, 8]. Our findings from clinical acute allograft rejection suggest that in this setting, primary cilia remain a factor in the modulation of signaling pathways in the epithelial layer. Primary cilia facilitate Hh signaling [2] but suppress canonical Wnt signaling [9]. We speculate that increases in primary cilium length following the onset of acute rejection injury represent an epithelial repair related program acting to re-establish suppression of the canonical Wnt pathway while simultaneously promoting Hh signaling. We also saw that Smo accumulated in the elongated renal primary cilia from a mouse model of acute injury, but not in primary cilia from uninjured control tissue. This suggests that hedgehog signaling is activated in epithelial cells to repair the damage to the kidney. Studies of renal cystic disease point to a role for Hh signaling in driving epithelial proliferation [10, 11], an important component of repair in the injured kidney.

A study by von Toerne et al. [5] investigating signaling pathways involved in a rat model of chronic renal allograft rejection reported that changes in Wnt and Hh signaling are linked to chronic allograft rejection causing the loss of primary cilia on epithelial cells. The primary cilium has previously been implicated in the regulation of these pathways and immunostaining presented supports the authors’ observation that primary cilia are lost during chronic rejection. However other data shown is not consistent with the idea that the loss of primary cilia on the epithelium is modifying the regulation of Hh signaling in the manner reported. Primary cilia are typically required for Hh signaling in vertebrates [2] but data shows that two key Hh target genes (Ptch1 and Gli1) are upregulated in chronic renal allograft rejection, suggesting the activation of the Hh pathway. The kidney is a complex organ with many cells types, in addition to epithelial cells, that may contribute to the net modification of Hh signaling as observed during chronic renal allograft rejection.

An important distinction may be that von Toerne et al. [5] studied a model of chronic renal allograft rejection, while we examined acute renal allograft rejection controlled by immunosuppressive drugs. It is possible that as acute rejection progresses to chronic rejection, the repair program may fail and subsequent loss of cells with an epithelial phenotype may reduce the production of apical primary cilia in the tubule and duct as observed by von Toerne et al. [5].

## Limitations

Two biopsy series (one acute cellular rejection and one antibody-mediated rejection) were examined and ciliary smoothed was the only marker of hedgehog activation studied.

## Data Availability

The datasets used and/or analysed during the current study are available from the corresponding authors on reasonable request.

## Abbreviations

Wnt: wingless
Smo: smoothened
Ptch1: patched
IR: ischaemia reperfusion
